# Circulating sortilin level as a potential biomarker for coronary atherosclerosis and diabetes mellitus

**DOI:** 10.1186/s12933-017-0568-9

**Published:** 2017-07-20

**Authors:** Tae Jung Oh, Chang Ho Ahn, Bo-Rahm Kim, Kyoung Min Kim, Jae Hoon Moon, Soo Lim, Kyong Soo Park, Cheong Lim, HakChul Jang, Sung Hee Choi

**Affiliations:** 1 Department of Internal Medicine, Seoul National University College of Medicine, Seoul, South Korea; 2 Department of Internal Medicine, Seoul National University Bundang Hospital, 300, Gumi-dong, Bundang-gu, Seongnam, 463-070 South Korea; 3 Department of Thoracic and Cardiovascular Surgery, Seoul National University College of Medicine, Seoul, South Korea; 4 Department of Thoracic and Cardiovascular Surgery, Seoul National University Bundang Hospital, Seongnam, South Korea

**Keywords:** Coronary artery disease, Diabetes mellitus, Sortilin, Proneurotensin, Biomarker

## Abstract

**Context:**

A previous genome-wide association study showed that a genetic variant of sortilin was associated with the risk of coronary artery disease (CAD). However, the role of circulating sortilin is still unknown. We investigated the potential role of plasma sortilin as a biomarker for CAD and diabetes mellitus.

**Methods:**

We enrolled statin-naïve subjects with CAD (n = 31) who underwent coronary artery bypass surgery and control subjects (n = 116) who were free from CAD as evaluated by coronary CT angiography. The presence of diabetes mellitus was evaluated and plasma sortilin levels were measured with a commercial ELISA kit.

**Results:**

Plasma sortilin levels were higher in subjects with CAD and subjects with diabetes mellitus than in those without CAD or diabetes mellitus. Subjects in the highest sortilin tertile group were older and had higher glucose and HbA1c levels, but lipid profiles in the three tertile groups were comparable. Multivariable logistic regression analysis revealed that sortilin levels were independently associated with CAD. In addition, the receiver operating characteristic curve analysis showed that plasma sortilin levels could identify the presence of CAD or diabetes mellitus.

**Conclusions:**

Elevated circulating sortilin levels are associated with CAD and diabetes mellitus and can be used as a biomarker of both diseases in statin-naïve subjects.

## Background

The number of adults with diabetes mellitus has nearly quadrupled in the USA over the past 30 years [1]. In addition, it is estimated that the global prevalence of diabetes mellitus will increase by 54% from 2010 to 2030 [2]. In Korea, the prevalence of diabetes mellitus is also increasing and the last national survey showed that the prevalence among adults 30 years or older is 13.7% [3]. However, the majority of individuals with diabetes are not aware of their condition and the rate may be up to 30% [3]. Therefore, biomarkers for diabetes mellitus should be developed to improve the identification of high-risk subjects.

In patients with diabetes mellitus, the leading cause of death is cardiovascular disease, even though the rates of cardiovascular disease declined between 1990 and 2010 [4]. It is unquestionable that diabetes mellitus is the most important risk factor for cardiovascular disease [5]. In this context, a biomarker for diabetes mellitus could be used for the prediction of CAD and vice versa. In fact, there are common shared biomarkers for CAD and diabetes mellitus [6, 7]. These biomarkers are useful for distinguishing patients at high risk for CAD [3, 8] and may be good candidates as therapeutic targets. In this context, investigation of biomarkers for both CAD and diabetes mellitus has important clinical implications.

The Wellcome Trust Case Control Consortium study showed that a single nucleotide polymorphism at the 1p13 locus was associated with the risk of CAD [9]. Subsequent studies demonstrated that the *SORT1* gene was associated with hepatic lipid metabolism [10] and low-density lipoprotein (LDL)-cholesterol levels [11]. The *SORT1* gene encodes sortilin, which belongs to the mammalian vacuolar protein sorting 10 protein domain family [12]. Sortilin is located mainly within cells in sites such as the trans-Golgi network, and it is also located in the cell membrane [12] and systemic circulation [13]. Few studies have reported that soluble sortilin and the propeptide fragment of its ligand, proneurotensin, were associated with cardiovascular risk factors [14, 15]. Ogawa demonstrated that soluble sortilin levels were positively associated with cardiovascular risk factors, but that soluble sortilin levels were lower in patients with CAD than controls [14]. In another study from the Framingham Heart Study, increased circulating proneurotensin was associated with a higher rate of cardiovascular events [15]. Therefore, there is some discrepancy between the two studies of prediction of CVD using these two circulating molecules related to sortilin. In this study, we measured circulating sortilin levels, and tested whether the measurement of circulating sortilin can be used as a biomarker of both CVD and diabetes mellitus.

## Methods

### Study population

We enrolled subjects with and without confirmed CAD. The subjects with CAD were recruited from the Thoracic Surgery Department of the Seoul National University Bundang Hospital (SNUBH). They underwent coronary artery bypass graft (CABG) surgery because of acute myocardial infarction, unstable angina, or stable angina with three-vessel disease. The subjects that were free from CAD, as evaluated by coronary CT angiography, were recruited from the outpatient clinic of Endocrinology Department at the same hospital. We excluded subjects who had been treated with statins within the previous 3 months or whose medical records were not available. A total of 31 subjects with CAD and 116 subjects without CAD were enrolled from May 2012 to January 2015. Of these, 43 (29.3%) subjects were diagnosed with diabetes mellitus. The study was approved by the Institutional Review Board of the SNUBH (IRB No B1203/147-006) and all subjects gave informed consent.

### Medical history and diagnosis

We defined CAD as acute myocardial infarction or angina pectoris that needed to be treated with CABG surgery. The definition of normal coronary arteries was the absence of luminal stenosis of ≥50% with a coronary artery calcium score <10. Diabetes mellitus was defined as a fasting plasma glucose ≥126 mg/dl and/or HbA1c ≥6.5% or treatment with antidiabetes medication. Medical records were reviewed and medical history and clinical characteristics such as age, sex, body weight, height, systolic and diastolic blood pressure were obtained. Biochemical analysis was performed immediately before surgery or within 3 months of coronary CT angiography. Nonfasting blood samples were obtained and centrifuged at 3000 rpm for 10 min at 4 °C. The plasma was stored at −80 °C until analysis.

### Biochemical assays

The plasma levels of glucose and HbA1c were measured by the hexokinase method and high-performance liquid chromatography, respectively. Total cholesterol, triglyceride, high-density lipoprotein (HDL)-cholesterol, and LDL-cholesterol levels were measured by relevant enzymatic assays and the glycerol-3-phosphate oxidase peroxide method. The high-sensitive C-reactive protein (hs-CRP) was measured by latex-enhanced turbidometric immunoassay (Denka Seiken Co., Tokyo, Japan). These laboratory analyses were performed by the central laboratory of SNUBH. Circulating plasma sortilin was measured using a commercial enzyme-linked immunosorbent assay (ELISA) (Cusabio ELISA kits, Cosmo Bio, Carlsbad, CA, USA). This ELISA kit can detect sortilin levels of 46.88–3000 pg/ml with a coefficient of variation <8% for intra-assay precision and <10% for interassay variation.

### Statistical analysis

Continuous variables are presented as mean and standard deviation and categorical variables are presented as percentages. The differences between two groups were analyzed by 2-tailed Student *t* test or nonparametric *t* test and age-adjusted analysis of covariance. Categorical variables were compared using the Chi square test. Comparison between the three tertile groups was performed using one-way analysis of variance (ANOVA) followed by least significant difference (LSD) post hoc analysis. A log transformation was applied to glucose, HbA1c, triglyceride, hs-CRP, and sortilin levels prior to performing ANOVA and logistic regression analysis. The association with CAD or diabetes mellitus was evaluated by univariable and multivariable logistic regression analysis. The area under the receiver-operating characteristics (ROC) curve was calculated to test its predictive discrimination of CAD. The optimal cut-off value was determined using the maximum sum of sensitivity and specificity based on the Youden-Index. Statistical analysis was performed using IBM SPSS Statistics for Windows, version 20.0 (IBM Corp., Armonk, NY, USA). For all tests, *P* < 0.05 was considered to be significant.

## Results

**Fig. 1:**
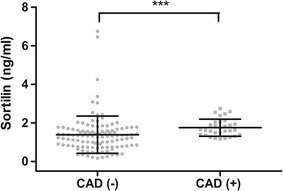
Circulating sortilin levels depending on the existence of coronary artery disease. Data are shown as mean ± SD. ***P < 0.001 using a nonparametric *t* test

The clinical and biochemical characteristics of subjects with or without CAD are shown in Table 1. Subjects with CAD were older and had higher systolic blood pressure and blood glucose levels than their counterparts. They showed lower HDL- and LDL-cholesterol levels than subjects without CAD and included a higher proportion of subjects diagnosed with diabetes mellitus, hypertension, and dyslipidemia. Circulating plasma sortilin levels were higher in subjects with CAD than those without CAD (Fig. 1). The significance remained after age adjustment. Two subjects that had extreme values for sortilin (6.47 and 6.75 ng/ml) did not have CAD. However, when we eliminated these two outliers the study results were not changed. Overall, 26 of 31 subjects with CAD took aspirin. We did not see any significant difference in circulating sortilin levels between aspirin users and non-users (1.72 ± 0.45 vs. 1.86 ± 0.42 ng/ml, *P* = 0.548).

**Table 1:** Clinical and biochemical characteristics of study participants classified according to coronary artery disease

	Total subjects	CAD (−), n = 116	CAD (+), n = 31	*P* value	*P* value*
Age (years)	55.5 ± 11.0	52.9 ± 9.2	65.4 ± 11.8	<0.001	N/a
Men (%)	69.4	66	84	0.052	N/a
Body weight (kg)	65.3 ± 11.0	65.8 ± 11.5	63.3 ± 8.60	0.203	0.886
BMI (kg/m^2^)	23.9 ± 2.8	24.1 ± 2.9	23.4 ± 2.5	0.213	0.587
SBP (mmHg)	122.2 ± 17.0	120.5 ± 16.4	128.6 ± 18.0	0.019	0.015
DBP (mmHg)	73.7 ± 11.7	74.1 ± 12.2	72.3 ± 9.5	0.438	0.633
Glucose (mg/dl)	107.0 ± 1.4	102.0 ± 30.9	161.0 ± 77.0	<0.001	<0.001
HbA1c (%)	6.2 ± 1.2	6.2 ± 1.4	6.9 ± 1.5	0.004	0.068
TC (mg/dl)	194.0 ± 40.3	202.1 ± 35.3	164.0 ± 43.9	<0.001	<0.001
LDL-C (mg/dl)	110.1 ± 26.8	112.6 ± 26.0	100.6 ± 28.4	0.026	0.097
HDL-C (mg/dl)	49.1 ± 12.3	52.0 ± 11.7	38.1 ± 7.2	<0.001	<0.001
TG (mg/dl)	132.7 ± 66.9	131.9 ± 67.4	135.6 ± 65.9	0.623	0.345
hs-CRP (mg/dl)	0.28 ± 0.71	0.11 ± 0.22	0.87 ± 1.26	<0.001	<0.001
DM (%)	29.3	19.8	64.5	<0.001	N/a
HTN (%)	53.7	43.1	93.5	<0.001	N/a
Dyslipidemia (%)	37.4	28.4	71.0	<0.001	N/a

Data are expressed as mean ± SD, or %. *CAD* coronary artery disease, *DM* diabetes mellitus, *BMI* body mass index, *SBP* systolic blood pressure, *DBP* diastolic blood pressure, *TC* total cholesterol, *LDL*-*C* LDL cholesterol, *HDL*-*C* HDL cholesterol, *TG* triglyceride, *HTN* hypertension

*P* values for student’s *t* test or nonparametric t test or Chi square test

* *P* values for age-adjusted ANCOVA

Next, we compared the variables in subjects with or without diabetes mellitus (Table 2). The subjects with diabetes mellitus showed similar clinical deterioration to the subjects with CAD. The sortilin levels were significantly higher in subjects with diabetes mellitus compared with those without diabetes mellitus (1.57 ± 0.54, and 1.34 ± 1.0 ng/ml, *P* = 0.006).

**Table 2:** Clinical and biochemical characteristics and sortilin levels in subgroup without coronary artery disease according to their diabetes mellitus status

	DM (−), n = 93	DM (+), n = 23	*P* value
Age (years)	52.5 ± 8.3	54.5 ± 12.0	0.444
Men (%)	31.2	47.8	0.148
Body weight (kg)	65.7 ± 11.4	66.0 ± 12.4	0.916
BMI (kg/m^2^)	23.8 ± 2.8	25.1 ± 2.9	0.058
SBP (mmHg)	120.2 ± 16.0	121.7 ± 18.2	0.696
DBP (mmHg)	73.8 ± 12.3	75.3 ± 11.8	0.614
Glucose (mg/dl)	90.8 ± 12.9	147.3 ± 40.3	<0.001
HbA1c (%)	5.7 ± 0.3	8.5 ± 1.8	<0.001
TC (mg/dl)	202.6 ± 34.5	200.1 ± 39.2	0.764
LDL-C (mg/dl)	111.2 ± 24.3	118.3 ± 31.8	0.243
HDL-C (mg/dl)	52.6 ± 12.0	49.6 ± 10.3	0.272
TG (mg/dl)	129.0 ± 67.9	143.3 ± 65.5	0.255
hs-CRP (mg/dl)	0.17 ± 0.52	0.56 ± 0.98	<0.001
Sortilin (ng/ml)	1.34 ± 1.0	1.57 ± 0.54	0.006
HTN (%)	61.1	41.2	0.008
Dyslipidemia (%)	71.0	73.9	0.999

Data are expressed as mean ± SD, or %. *CAD* coronary artery disease, *DM* diabetes mellitus, *BMI* body mass index, *SBP* systolic blood pressure, *DBP* diastolic blood pressure, *TC* total cholesterol, *LDL*-*C* LDL cholesterol, *HDL*-*C* HDL cholesterol, *TG* triglyceride, *HTN* hypertension

*P* values for student’s t test or nonparametric t test or Chi square test

Table 3 shows the general characteristics of study participants grouped according to sortilin tertiles. The mean age of subjects in tertile 1 was lower than those in tertiles 2 and 3. Body weight, blood pressure, and lipid profiles were comparable between the three tertile groups. However, blood glucose and HbA1c levels were higher in subjects in tertiles 2 and 3 than in subjects in tertile 1. In addition, more subjects in tertile 3 were diagnosed as having diabetes mellitus and CAD.

**Table 3:** General characteristics of patients by tertiles of circulating sortilin level

	Tertiles of circulating sortilin levels			*P* value
	Tertile 1 (<1.17 ng/ml) n = 49	Tertile 2 (1.17–1.59 ng/ml) n = 49	Tertile 3 (≥1.60 ng/ml) n = 49	
Age (years)	50.4 ± 6.5^a^	60.0 ± 9.9^b^	56.2 ± 13.4^b^	<0.001
Men (%)	73.5	67.3	67.3	0.750
Body weight (kg)	67.4 ± 10.2	64.2 ± 11.1	64.2 ± 11.5	0.249
BMI (kg/m^2^)	24.1 ± 2.3	23.9 ± 3.1	23.8 ± 3.0	0.890
SBP (mmHg)	119.9 ± 16.3	122.8 ± 15.9	124.0 ± 18.8	0.473
DBP (mmHg)	73.8 ± 11.7	73.9 ± 10.5	73.5 ± 12.9	0.985
Glucose (mg/dl)	94.0 ± 1.3^a^	113.3 ± 1.4^b^	115.1 ± 1.5^b^	0.003
HbA1c (%)	5.9 ± 1.1^a^	6.3 ± 1.2^a^	6.6 ± 1.3^b^	0.011
TC (mg/dl)	202.2 ± 34.9	191.7 ± 41.5	188.2 ± 43.5	0.206
LDL-C (mg/dl)	111.1 ± 22.5	109.9 ± 25.3	109.3 ± 32.3	0.946
HDL-C (mg/dl)	51.5 ± 13.7	49.7 ± 10.9	46.1 ± 11.9	0.082
TG (mg/dl)	122.0 ± 1.6	110.8 ± 1.7	118.9 ± 1.7	0.631
hs-CRP (mg/dl)	0.10 ± 0.15	0.28 ± 0.56	0.49 ± 1.09	0.015
DM (%)	8.2	34.7	44.9	<0.001
CAD (%)	0	26.5	36.7	<0.001
HTN (%)	14.3	75.5	71.4	<0.001
Dyslipidemia (%)	30.6	32.7	49.0	0.120

Data are expressed as mean ± SD, or %. *BMI* body mass index, *SBP* systolic blood pressure, *DBP* diastolic blood pressure, *TC* total cholesterol, *LDL*-*C* LDL cholesterol, *HDL*-*C* HDL cholesterol, *TG* triglyceride, *DM* diabetes mellitus, *CAD* coronary artery disease, *HTN* hypertension

Log-transformation was used for variables (glucose, HbA1c, triglyceride, hs-CRP, and sortilin) before statistical analysis. *P* values for one-way analysis of variance (ANOVA)

^a,b^ The data with different superscript letters are significantly different

As shown in Table 4, univariable logistic regression analysis revealed that age, systolic blood pressure, log glucose, log HbA1c, total cholesterol, HDL-cholesterol, LDL-cholesterol, log hs-CRP, and log sortilin were associated with CAD. We included these variables in multivariable logistic regression analysis but omitted log glucose, and total cholesterol to avoid collinearity. Multivariable logistic regression analysis indicated that age, HDL-cholesterol, log hs-CRP, and log sortilin were independently associated with the presence of CAD.

**Table 4:** Univariable and multivariable logistic regression analyses for coronary artery disease

	Association with presence of CAD			
	Single		Multiple	
	OR (95% CI)	*P* value	OR (95% CI)	*P* value
Age (years)	1.14 (1.08–1.20)	<0.001	1.08 (1.00–1.17)	0.04
BMI (kg/m^2^)	0.91 (0.79–1.06)	0.213	–	–
SBP (mmHg)	1.03 (1.00–1.05)	0.021	1.03 (0.98–1.08)	0.262
DBP (mmHg)	0.99 (0.85–1.02)	0.435	–	–
Log glucose (mg/dl)	41.69 (9.19–189.12)	<0.001	–	–
Log HbA1c (%)	10.06 (1.54–65.87)	0.016	0.12 (0.02–7.61)	0.124
TC (mg/dl)	0.97 (0.96–0.99)	<0.001	–	–
Log TG (mg/dl)	1.13 (0.52–2.48)	0.757	–	–
HDL-C (mg/dl)	0.86 (0.81–0.91)	<0.001	0.82 (0.72–0.93)	0.002
LDL-C (mg/dl)	0.98 (0.97–1.00)	0.028	1.01 (0.98–1.05)	0.513
Log hs-CRP (mg/dl)	2.64 (1.80–3.88)	<0.001	2.27 (1.28–4.04)	0.005
Log sortilin (ng/ml)	4.17 (1.68–10.35)	0.002	5.29 (1.06–26.51)	0.043

*CAD* coronary artery disease, *DM* diabetes mellitus, *BMI* body mass index, *SBP* systolic blood pressure, *DBP* diastolic blood pressure, *TC* total cholesterol, *TG* triglyceride, *HDL*-*C* HDL cholesterol, *LDL*-*C* LDL cholesterol

Log-transformation was used for variables (glucose, HbA1c, triglyceride, hs-CRP, and sortilin) before statistical analysis. *P* values for univariable or multivariable logistic regression analysis

**Fig. 2:**
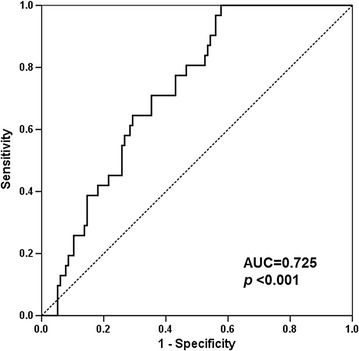
ROC curve analysis of the ability of sortilin to predict the presence of coronary artery disease

The ability of the area under the receiver operating characteristic (ROC) curve based on sortilin levels to predict the presence of CAD was 0.725 (Fig. 2). The optimal cut-off values of sortilin were 1.35 ng/ml (80.6% sensitivity and 53.0% specificity) to detect CAD. We performed same analysis in the subgroup without CAD. In this analysis we found that the area under ROC curve for sortilin levels to predict the presence of diabetes mellitus was 0.681, and the asymptotic significance was 0.007.

## Discussion

This is the first study to demonstrate that circulating sortilin levels measured by commercial ELISA are associated with both CAD and diabetes mellitus. Subjects with CAD or diabetes mellitus had higher sortilin levels than control subjects, and plasma sortilin levels were independently associated with CAD. In subgroup of subjects free from CAD, circulating sortilin levels were also associated with the presence of diabetes mellitus. These results demonstrated that sortilin levels can be used as a biomarker for CAD and diabetes mellitus, further studies are required to validate these results and to determine the relationship between sortilin and glucose metabolism.

Sortilin is one of three neurotensin receptors, neurotensin receptor-3 [16]. The level of proneurotensin, the propeptide of neurotensin, was reported to be related to the risk of incident diabetes mellitus in a prospective cohort study [17]. In this cohort, individuals with higher circulating proneurotensin had a higher incidence of diabetes mellitus, with a hazard ratio of 1.28 (95% confidence interval 1.07–1.50, *P* = 0.003). In addition, in another human study, plasma proneurotensin levels were higher in insulin-resistant obese subjects [18]. In our study, plasma levels of sortilin, a receptor for neurotensin, were related to diabetes mellitus and insulin resistance. Therefore, sortilin/neurotensin signaling might play an important role in insulin signaling. One possible explanation for this is suggested by the role of sortilin in glucose transporter-4 translocation in adipocytes [19]. Furthermore, neurotensin deficient mice were resistant to high-fat diet-induced obesity, and neurotensin decreases AMP-activated protein kinase activity via sortilin [18]. Our study results add new evidence for the link between sortilin and insulin signaling and glucose regulation.

Our results are noteworthy because we included subjects using strict criteria that minimized possible bias. First, we excluded subjects taking statin therapy. A previous study showed that treatment with pitavastatin or pravastatin for 8 months significantly decreased the circulating sortilin levels compared with baseline by 8% for pitavastatin and 16% for pravastatin [20]. Therefore, statin therapy has an impact on circulating sortilin levels, and we eliminated this bias by including only statin-naïve subjects. Second, we defined a normal coronary artery based on coronary CT angiography, a test that has a high negative predictive value [21]. Therefore, our data were not confounded by uncertainty about the presence of CAD, in contrast to other studies that define the absence of CAD from the subjects’ medical history. Third, we analyzed the data by several statistical methods, and the results were consistent.

Genome-wide association studies have provided good insight into the relationship between lipid metabolism and CAD [22, 23]. From these studies the cardiovascular phenotype of genetic variants seems to depend on ethnicity or the presence of diabetes mellitus. Variants in the *SORT1* locus also showed an association with lipid metabolism and CAD [24]. However, the effect of this genetic variant on blood sortilin levels has not been clarified. In contrast to our data, a Japanese study [14] showed that the soluble sortilin levels were lower in subjects with CAD than in controls. The Japanese study used a different type of assay to measure the sortilin levels, and the majority of study subjects were being treated with statins. Therefore, when we used sortilin as a biomarker, we made an effort to standardize the measurement of blood sortilin levels and elucidate the impact of various medications on its levels.

Sortilin is expressed widely, including in the nervous system, liver, adipose tissue, and in immune cells including macrophages [25]. Whole-body sortilin-knockout mice had decreased plasma HDL- and LDL-cholesterol levels accompanied by attenuation of atherosclerotic lesions [10] and vascular calcification [26]. Similarly, macrophage sortilin-deficient mice showed reduced atherosclerotic lesions because of reduced LDL uptake by macrophages [27]. In contrast, serum LDL-cholesterol levels were decreased rather than increased after overexpression of sortilin in the liver using adenovirus [28]. This study used sortilin molecules including the C-terminus, and this form of sortilin is likely to include the transmembrane region. This type of sortilin enhanced LDL clearance via an LDL receptor-independent pathway. A subsequent study demonstrated that hepatic sortilin reduces hepatic apolipoprotein B secretion and increases LDL catabolism [29]. In contrast, another study using sortilin without the C-terminal region enhanced hepatic very low density lipoprotein (VLDL), leading to increased LDL levels in the blood [10]. Therefore, the role of sortilin in lipid metabolism seems to be differ according to its structure, location, and target tissue. To our knowledge, there are no previous studies of the correlation between tissue and circulating levels of sortilin. In our study, we confirmed that higher circulating sortilin levels were related to an increased risk of CAD. However, we did not see any significant correlation between circulating sortilin levels and LDL-cholesterol or hepatic sortilin content. In the case of LDL-cholesterol, one type of sortilin can reduce VLDL secretion, while the other type of sortilin can enhance internalization of LDL-cholesterol. In addition, the exact role of circulating sortilin, the type measured in our study, is not known. There may be mechanisms other than lipid metabolism that contribute to the association between circulating sortilin and CAD. Interestingly, in the subgroup of subjects without CAD the insulin resistance calculated by HOMA-IR was positively correlated with circulating sortilin levels (Spearman’s ρ = 0.513, *P* = 0.015). Insulin resistance could be a link between sortilin, atherosclerosis and diabetes mellitus. Further mechanistic studies are needed to confirm this hypothesis.

## Limitations

Our study has several limitations. First, this is a cross-sectional study, so we could not predict future development of CAD and diabetes mellitus. The suggested cut-off value of sortilin for discriminating CAD had relatively low specificity, so we were unable to diagnose CAD solely based on blood sortilin level. A large-scale prospective cohort study is needed to confirm the usefulness of circulating sortilin for screening of CAD. In addition, usefulness of sortilin to screen or predict diabetes mellitus is doubtful, because measuring plasma glucose level is more practical than assaying for sortilin. Second, we did not analyze the sortilin levels according to disease severity, e.g., comparing subclinical and clinical atherosclerosis, or prediabetes to overt diabetes. Therefore, we cannot generalize our data to a broad range of CAD including subclinical atherosclerosis. Third, even though we adjusted for the age difference between groups, the age discrepancy could be a confounder. Furthermore, statistical analysis for DM was not feasible because of the small number of subjects without diabetes mellitus who underwent CABG. Fourth, we did not investigate the influence of antidiabetic and antihypertensive medication. Finally, we had no data concerning the presence of single nucleotide polymorphisms of sortilin.

## Conclusions

Circulating sortilin level was associated with both CAD and diabetes mellitus. Elevated circulating sortilin levels were an independent risk factor for CAD and could be a useful biomarker for these two diseases, especially in statin-naïve subjects.

## Abbreviations

ANOVA: analysis of variance
CABG: coronary artery bypass graft
CAD: coronary artery disease
LSD: least significant difference
